# Early lean mass sparing effect of high-protein diet with excess leucine during long-term bed rest in women

**DOI:** 10.3389/fnut.2022.976818

**Published:** 2022-11-24

**Authors:** Pierandrea Vinci, Filippo Giorgio Di Girolamo, Alessandro Mangogna, Filippo Mearelli, Alessio Nunnari, Nicola Fiotti, Mauro Giordano, Marie-Pierre Bareille, Gianni Biolo

**Affiliations:** ^1^Department of Medical Surgical and Health Sciences, Medical Clinic, Cattinara Hospital, University of Trieste, Trieste, Italy; ^2^Hospital Pharmacy, Cattinara Hospital, Azienda Sanitaria Universitaria Giuliano Isontina, Trieste, Italy; ^3^Institute for Maternal and Child Health, Istituto di Ricovero e Cura a Carattere Scientifico (IRCCS) Burlo Garofolo, Trieste, Italy; ^4^Department of Advanced Medical and Surgical Sciences, University of Campania L. Vanvitelli, Naples, Italy; ^5^Institute of Space Physiology and Medicine (MEDES), Toulouse, France

**Keywords:** lean body mass, high-protein diet, branched-chain amino acids, leucine, long-term bed rest

## Abstract

Muscle inactivity leads to muscle atrophy. Leucine is known to inhibit protein degradation and to promote protein synthesis in skeletal muscle. We tested the ability of a high-protein diet enriched with branched-chain amino acids (BCAAs) to prevent muscle atrophy during long-term bed rest (BR). We determined body composition (using dual energy x-ray absorptiometry) at baseline and every 2-weeks during 60 days of BR in 16 healthy young women. Nitrogen (N) balance was assessed daily as the difference between N intake and N urinary excretion. The subjects were randomized into two groups: one received a conventional diet (1.1 ± 0.03 g protein/kg, 4.9 ± 0.3 g leucine per day) and the other a high protein, BCAA-enriched regimen (1.6 ± 0.03 g protein-amino acid/kg, 11.4 ± 0.6 g leucine per day). There were significant BR and BR × diet interaction effects on changes in lean body mass (LBM) and N balance throughout the experimental period (repeated measures ANCOVA). During the first 15 days of BR, lean mass decreased by 4.1 ± 0.9 and 2.4 ± 2.1% (*p* < 0.05) in the conventional and high protein-BCAA diet groups, respectively, while at the end of the 60-day BR, LBM decreased similarly in the two groups by 7.4 ± 0.7 and 6.8 ± 2.4%. During the first 15 days of BR, mean N balance was 2.5 times greater (*p* < 0.05) in subjects on the high protein-BCAA diet than in those on the conventional diet, while we did not find significant differences during the following time intervals. In conclusion, during 60 days of BR in females, a high protein-BCAA diet was associated with an early protein-LBM sparing effect, which ceased in the medium and long term.

## Introduction

Physical inactivity or bed rest (BR) frequently occurs in patients affected by several diseases, but also during physiologic aging (1–3). Muscle inactivity leads to muscle atrophy, loss of bone mineral density and decreased insulin sensitivity (2, 4–10). Beyond its well-known functional-motor structural role, skeletal muscle represents a homeostatic “organ,” fundamental for the maintenance of whole-body metabolic control (11). Indeed, skeletal muscle mass regulates the amino acid balance of organs and tissues, as well as the availability of nutrients and metabolic precursors (11–13). Moreover, it has a central role in maintaining the general energy balance, through complex interplay mechanisms, and contributes to preserve the individual’s state of health and quality of life (11).

Together, with physical activity, nutrition has a fundamental role in homeostatic regulation and maintenance of muscle mass and function (14–16). Among nutrients, essential amino acids, occupy a central place, linked not only to the formation of proteins, but also to the control of numerous and complex subcellular pathways, fundamental for maintaining cellular energy balance and, ultimately, for survival itself (17–19). The action of essential amino acids on muscle, in addition to being of a direct type on specific regulatory factors (i.e., mammalian target of rapamycin, mTOR, complex 1), is also mediated by hormonal control (i.e., insulin, glucagon, cortisol), involved in anabolic and catabolic processes, through the regulation of transcriptional sequences (19, 20). Evidence indicates that high-protein diets and essential amino acid supplementation may ameliorate muscle protein loss in healthy volunteers during experimental BR (21, 22). In addition, protein/amino acid intake has been reported to modulate insulin signaling and β-cell function in “*in vivo*” experiments (23, 24). Branched-chain amino acids (BCAAs), i.e., leucine, valine, and isoleucine are essential amino acids, exhibiting selective effects not only on stimulation of muscle protein synthesis, but also on insulin mediated glucose uptake, moreover they play an important role in several metabolic and signaling functions, particularly *via* activation of the mTOR signaling pathway (25, 26). Among BCAAs, the most noteworthy effects have been observed with leucine (26). In particular, leucine: (a) is a constituent of proteins (27); (b) regulates protein synthesis translation initiation in skeletal muscle (28–30); (c) modulates insulin/phosphoinositide 3-kinase (PI3K) signal cascade (31); (d) is a fuel for skeletal muscle cells (32); (e) is a primary nitrogen donor for the production of alanine and glutamine in skeletal muscle (33); (f) modulates the pancreatic β-cell insulin release (34). All these diverse metabolic roles allow leucine to influence the rate of muscle protein synthesis, insulin secretion and glucose homeostasis (25, 35). Evidence indicates that leucine alone may exert and anabolic response (36), while no such data exists for isoleucine or valine, although isoleucine could potentially increase muscle growth by up-regulating the protein expression of GLUT1 and GLUT4 in muscle (26).

The aim of the present study was to assess the effects of a high-protein diet enriched with BCAAs on progression of lean body mass (LBM) atrophy over 2-months experimental BR in healthy young female volunteers as an experimental model of long-term physical inactivity. LBM was assessed approximately every 2 weeks by dual-energy X-ray absorptiometry (DXA). Whole-body protein kinetics were evaluated by nitrogen (N) balance before and during BR. Healthy volunteers who underwent a prolonged BR represent a good model to investigate the effects of muscle unloading on physiological functions (37).

## Materials and methods

### Subjects and study design

Sixteen medically and psychologically healthy females, aged 25–40 years (32 ± 4 years), participated in the Women’s International Space Simulation for Exploration (WISE)—2005 BR study. The study was completed in two campaigns (February 2005–May 2005, September 2005–December 2005). Non-smoking volunteers were recreationally active but athletically untrained, thus competitive athletes were excluded from the study. Included volunteers met the following criteria: a body mass index between 20 and 25 kg/m^2^, regular menstrual cycles, no intake of oral contraceptives in the 2 months before the study, no family history of chronic diseases or psychiatric disorders, and absence of musculoskeletal, orthopedic, blood clotting, and cardiovascular disorders. Mean weight and height of the subjects were 59 ± 4 kg and 166 ± 7 cm, respectively.

The procedures were conducted in accordance with the ethical principles stated in the Declaration of Helsinki 1964. The protocol was reviewed and approved by the local ethics committee (Comité Consultatif de Protection des Personnes dans la Recherche Biomédicale, CCPPRB—Toulouse 1: dossier n°1-04-16: 20/07/04—avis n°2: p.1), by the University of California, San Diego Institutional Review Board and by the Institutional Review Boards of the National Aeronautics and Space Administration, Johnson Space Center Committee for the Protection of Human Subjects. The whole study procedures was explained to the volunteers both verbally and in writing. All participants provided a written informed consent. The study was performed at the Institute for Space Medicine and Physiology in Toulouse, France. The whole study protocol is discussed in detail elsewhere (38, 39), briefly, it consisted in a long-term BR which included a 20-day ambulatory control period followed by 60 days of strict 6° head-down-tilt (HDT) BR (40). A 20-day ambulatory recovery period followed the BR period.

After a 20-day ambulatory adaptation to a controlled diet (i.e., conventional diet), subjects were randomized into two eucaloric diet groups (*n* = 8, each): a control group who continued the conventional diet and a protein group on a high protein diet enriched with BCAA. During the 20 days pre-BR (ambulatory period, AMB), resting metabolic rate was determined by indirect calorimetry using Deltatrac II (General Electric, Indianapolis, IN) according to the manufacturer. The prescribed caloric intake for the two groups was 140% of their resting metabolic rate during the pre-BR. During BR, energy intake was adjusted downward to 110% of resting metabolic rate both in the control (conventional diet) and protein (high protein-BCAA diet) groups. In the control group on the conventional diet, the prescribed daily protein intake was 1.1 ± 0.03 g⋅kg^–1^⋅day^–1^ of body weight; while, in the group on the high protein-BCAA diet, dietary protein content was increased to about 1.45 g⋅kg^–1^⋅day^–1^ and enriched with 0.15 g⋅kg^–1^⋅day^–1^ of BCAAs (leucine/valine/isoleucine = 2/1/1). Free BCAAs (3.6 g/day free leucine, 1.8 g/day free isoleucine, and 1.8 g/day free valine) were added as a supplement (Friliver, Bracco, Italy) during the three main meals. Thus, the total protein/amino acid daily intake for this group was of 1.6 ± 0.03 g⋅kg^–1^ body weight. To compensate for the additional increase in energy intake from protein in this group, carbohydrate content was reduced during the BR period. Fat mass was monitored every 15 days and maintained at basal levels by changing energy intake, if necessary. All subjects were always restricted to the HDT position except for mealtime when they were allowed to elevate on one elbow. Body weight, urine production, intake of fluids and body temperature were regularly monitored.

### Body mass and composition

Body weight was measured daily during pre-BR and during BR. Lean and fat mass were determined by DXA with a Hologic QDR 4,500 W, Software Version 11.1 (Hologic, Bedford, MA). At baseline, measurements were obtained twice, respectively, on days −14 and −2 before the start of BR in each group. In the ambulatory condition DXA measurements obtained at days −14 and −2 were averaged. DXA scans were performed four times during the 60-day BR, respectively, on days 15, 31, 43, and 60 (days BR 15, BR 31, BR 43, and BR 60). DXA scans were executed in the morning in the fasting state. The bladder was emptied before the scan. The subjects had nothing to eat or drink after dinner the night before.

### Nitrogen balance during the bed rest

Nitrogen (N) balance reflects the equilibrium between protein N intake and N losses to define anabolic and catabolic conditions of whole-body protein kinetics (i.e., difference between rates of synthesis and degradation). N balance is calculated as the difference between N intake and N losses. In this study, N balance was estimated from the difference between N protein/amino acid intake and urinary N excretion (41), with the addition of a constant value of 4 g/day to account for N losses from the skin and feces (41, 42). A total of 24-h urine samples were collected, aliquoted in 10 mL samples and at −20°C. Urinary N from these aliquots was measured by chemo-luminescence (Antek 7000, Antek Instruments, U.S.) in an accredited laboratory (Central Biochemical Laboratory, Université de Lyon, Lyon, France). Protein intake was assessed by entering all food eaten including all ingredients used to prepare complex recipes, into the Nutrilog Edition Expert software, version 2.0 (Marans, France). All food and leftovers were weighed individually. N balance [g] was then calculated daily according to the following equation: N balance = (24-h protein/BCAA intake/6.25) – (24-h urinary urea N + 4). In the ambulatory condition N balance of days −1 and −2 were averaged. During the BR period the average daily N balance values were calculated in the time intervals between consecutive LBM measurements. Values of N balance were expressed as mg/kg LBM per day.

### Data and statistical analysis

The numerical data are presented as means ± standard deviation (SD). The differences between pre-BR and during 60-days BR were analyzed by repeated-measures ANCOVA with time (AMB or BR days) and diet (conventional or high protein-BCAA diet) as the two factors using ambulatory values as covariates. *Post hoc* analysis was performed, when appropriate, by using paired *t*-test or Mann-Whitney test with Bonferroni’s adjustments. Levene’s test showed normal distribution and equal variance of ambulatory values of LBM, N intake, N excretion and N balance in the two groups. All *p*-values were considered significant when < 0.05. All analyses were performed using the IBM SPSS statistic 21 software (Version 21.0, SPSS Inc., Chicago, IL, USA).

## Results

Table 1 shows mean values of nutrient intakes in the AMB and BR periods in the conventional and high protein-BCAA diet groups. During BR, energy intake decreased by about 8% in both groups. Protein intake increased during BR by about 11 and 53% in the conventional and high protein-BCAA diet groups, respectively. Leucine intake increased by about 100% during the BR period in the high protein-BCAA diet group.

**TABLE 1:** Summary of dietary intake in ambulatory condition and during 60-days of bed rest.

			AMB	BR	*p* BR effect	*p* BR × diet
Energy intake	(kcal⋅kg^–1^⋅day^–1^)	Conventional diet	32.3 ± 3.1	29.3 ± 2.0*	<0.001	0.12
		High protein-BCAA diet	31.7 ± 1.1	29.9 ± 1.1*		
Carbohydrate intake	(% energy intake)	Conventional diet	58 ± 0.6	56 ± 0.8*	<0.001	<0.001
		High protein-BCAA diet	57 ± 0.6	50 ± 1.1*^§^		
Lipid intake	(% energy intake)	Conventional diet	30 ± 0.3	30 ± 0.6*	<0.001	<0.001
		High protein-BCAA diet	30 ± 0.3	28 ± 0.3*^§^		
Protein/amino acid intake	(% energy intake)	Conventional diet	13 ± 0.6	14 ± 0.6*	<0.001	<0.001
		High protein-BCAA diet	13 ± 0.6	21 ± 1.1*^§^		
	(g⋅kg^–1^⋅day^–1^)	Conventional diet	1.0 ± 0.03	1.1 ± 0.03*	<0.001	< 0.001
		High protein-BCAA diet	1.0 ± 0.03	1.6 ± 0.03*^§^		
Leucine intake	(g⋅day^–1^)	Conventional diet	5.2 ± 0.4	4.9 ± 0.3	<0.001	<0.001
		High protein-BCAA diet	5.7 ± 0.4	11.4 ± 0.6*^§^		

AMB, ambulatory; BR, bed rest. All data were expressed as means ± SD. All data were analyzed by repeated-measures ANCOVA with time (AMB or BR days) and diet (conventional or high protein-BCAA diet) as the two factors using AMB values as covariates. *Post hoc* analysis was performed, when appropriate, by using paired *t*-test with Bonferroni’s adjustment. *p < 0.05, BR vs. AMB. ^§^*p* < 0.05, high protein-BCAA vs. normal protein, conventional diet); SD, standard deviation.

Changes in body weight and composition during the 60-day BR are shown in Table 2. Body weight significantly decreased following BR. At the end of the BR period body weight decreased by 5.9 ± 1.1% in the conventional diet group and by 4.1 ± 1.7% in the high protein-BCAA diet group (*p* = 0.01). Fat mass did not change significantly during the BR period in both groups. Lean mass significantly decreased during the first 31 days of BR and did not change during the following 29 days of BR, both in the high protein-BCAA and the conventional diet groups. There were significant BR effect and BR × diet interaction on changes in LBM.

**TABLE 2:** Body composition in ambulatory condition and during 60-days of bed rest.

		AMB	BR 15 days	BR 31 days	BR 43 days	BR 60 days	*p* BR effect	*p* BR × diet
Total mass	Conventional diet	55.6 ± 3.9	54.1 ± 4.0*	52.9 ± 4.1*	52.6 ± 4.0*	52.3 ± 3.8	<0.001	0.02
	High protein-BCAA diet	61.0 ± 4.4	60.1 ± 3.9*	59.1 ± 4.0*	58.8 ± 4.0	58.5 ± 4.1		
Lean mass	Conventional diet	38.8 ± 3.0	37.2 ± 3.2*	36.1 ± 3.1*	36.1 ± 2.9	35.9 ± 2.9	0.008	0.012
	High protein-BCAA diet	42.8 ± 5.2	41.7 ± 4.2*	40.3 ± 4.6*	40.6 ± 4.3	39.8 ± 4.1		
Fat mass	Conventional diet	14.7 ± 3.8	14.7 ± 3.9	14.7 ± 3.8	14.3 ± 3.6	14.3 ± 3.5	0.57	0.08
	High protein-BCAA diet	15.9 ± 2.1	16.1 ± 2.0	16.5 ± 2.1	15.9 ± 2.0	16.4 ± 2.0		

Body composition measurement with dual-energy X-ray absorptiometry (DXA). Units are kg. Data are means ± SD. AMB, ambulatory; BR, bed rest. AMB values are means of measurements at –14 and –2 pre-bed rest days. All data were expressed as means ± SD. All data were analyzed by repeated-measures ANCOVA with time (AMB or BR days) and diet (conventional or high protein-BCAA diets) as the two factors using AMB values as covariates. *Post hoc* analysis was performed, when appropriate, by using paired *t*-test with Bonferroni’s adjustment. *p < 0.05, vs. the preceding AMB or BR day of assessment; SD, standard deviation.

**FIGURE 1:**
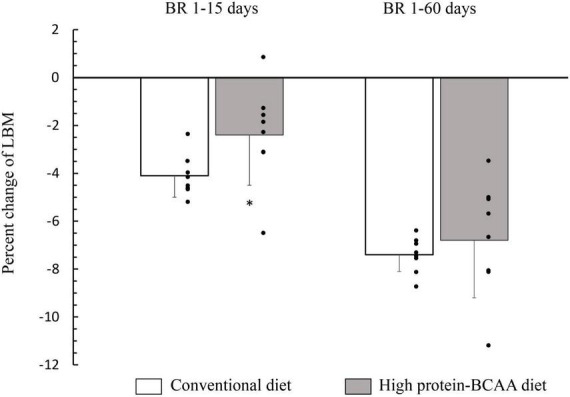
Effects of high protein-BCAA diet on lean body mass (LBM) during the first 15 days and during entire bed rest (BR) period of 60 days. All data were expressed as means ± SD. Individual data are additionally shown. All data were analyzed by repeated-measures ANCOVA with time (BR 1–15 days or BR 1–60 days) and diet (conventional or high protein-BCAA diet) as the two factors. BR effect, *p* < 0.001; BR × diet interaction, *p* = 0.036. *Post hoc* analysis was performed by using Mann-Whitney test with Bonferroni’s adjustment. *p < 0.05, high protein-BCAA vs. normal protein (conventional diet); SD, standard deviation.

After 15 days from the beginning of the BR, the decrease in lean mass was about twice greater in the conventional diet group than that in the high protein-BCAA diet group (Figure 1). Nonetheless, at the end of the 60 days of BR the total changes in LBM were similar in the two groups.

As shown in Table 3, during the BR period N intake and urine N excretion were 55 ± 8 and 52 ± 1% greater in subjects on the high protein-BCAA diet than in those on the conventional diet. There were significant BR effect and BR × diet interaction on N balance in both groups. Between the 1st and the 15th day of BR, N balance was significantly greater in the high protein-BCAA diet group than in the conventional diet group, while we did not find significant differences during the following time intervals.

**TABLE 3:** Nitrogen (N) intake, loss and balance.

		AMB	BR 1–15 days	BR 16–31 days	BR 32–43 days	BR 44–60 days	*p* BR effect	*p* BR × diet
N intake	Conventional diet	241 ± 17	234 ± 20	239 ± 20	240 ± 17	233 ± 11	<0.001	< 0.001
	High protein-BCAA diet	268 ± 31	359 ± 23*	370 ± 20*	373 ± 20*	363 ± 23*		
N loss	Conventional diet	276 ± 43	329 ± 19	327 ± 15	321 ± 16	322 ± 24	<0.001	< 0.001
	High protein-BCAA diet	309 ± 38	416 ± 30*	427 ± 21*	441 ± 21*	421 ± 28*		
N balance	Conventional diet	–35 ± 44	–95 ± 14	–88 ± 17	–81 ± 17	–89 ± 15	<0.001	0.01
	High protein-BCAA diet	–41 ± 44	–57 ± 19*	–56 ± 27	–68 ± 30	–58 ± 28		

AMB, ambulatory; BR, bed rest. Units are mg N × kg LBM^–1^ × d^–1^. All data were expressed as means ± SD. Values of N balance were not significantly different from zero (*p* > 0.15) in AMB conditions but were significantly negative (*p* < 0.01) in all BR conditions (paired *t*-test with Bonferroni’s adjustment). All data were analyzed by repeated-measures ANCOVA with time (AMB or BR days) and diet (conventional or high protein-BCAA diet) as the two factors using AMB values as covariates. *Post hoc* analysis was performed by using unpaired *t*-test with Bonferroni’s adjustment. *p < 0.05, high protein-BCAA vs. conventional diet. LBM, lean body mass; SD, standard deviation.

## Discussion

We have studied healthy young women during 60 days of BR in eucaloric conditions at different levels of protein intake. In the high protein-BCAA and conventional diet groups protein/amino acid intakes were about 1.6 and 1.1 g⋅kg^–1^⋅day^–1^, respectively. The high protein diet was enriched with 0.15 g⋅kg^–1^⋅day^–1^ of BCAAs with the following BCAA proportions, leucine/valine/isoleucine: 2/1/1. The diet high in protein and BCAA caused a slowing down of the loss of lean mass secondary to BR of about 42%, only in the first 15 days of inactivity. This lean mass saving effect was abolished in the later stages of BR. In fact, at the end of long-BR the total changes in LBM were similar in the two groups. The results of N balance agreed with changes in LBM.

The subjects were in energy balance during the whole study period as demonstrated by the absence of significant changes in fat mass. This finding is important as our previous studies showed that a positive or negative energy balance accelerated BR-induced muscle atrophy (43, 44). In fact, we have demonstrated that during 5 weeks of BR a positive energy balance was associated with greater loss of LBM and activation of the systemic inflammatory response and antioxidant defenses (44–48). Several pieces of literature provide evidence to support the role of inflammation in impairing muscle homeostasis with a consequent loss of skeletal muscle (49–51). We have also shown that a negative energy balance (hypocaloric nutrition) during 2 weeks of BR leads to a greatest wasting of LBM by increasing protein catabolism in the postabsorptive period and by impairing postprandial anabolic utilization of free amino acids (43).

Muscle atrophy depends on an imbalance between muscle protein synthesis and breakdown. It has been suggested that disuse atrophy in humans is caused mainly by a decreased basal protein synthesis, with no changes in protein degradation, at least in the early stages of BR or immobilization (52–54). In fact, the rate of basal protein synthesis declines immediately after unloading and stays at a suppressed level for the duration of the disuse (52–59). The most important role in the loss of muscle mass during a period of disuse has been explained elsewhere (60, 61) as a decline in both post-absorptive and postprandial muscle protein synthesis rates. This response now called anabolic resistance, is a state of diminished muscle protein synthesis, despite provision of an adequate amount of essential amino acids to elicit an appropriate synthesis response (54, 60–66). In addition, during limb immobilization and BR, a decreased post-absorptive muscle protein synthesis has been reported (55, 60, 62, 67, 68).

In our study, we speculated that the beneficial effect of a high protein-BCAA diet on LBM in the early phase of BR was due to leucine supplementation while maintaining an adequate intake of protein and of the other BCAAs, valine and isoleucine. In fact, several studies have shown that leucine, considered a strong stimulator of protein synthesis (30, 69, 70), has anabolic effects on protein metabolism by increasing the rate of protein synthesis and by decreasing the rate of protein degradation in resting human muscle (26, 30, 35, 71–73). On one hand, leucine increases muscle protein synthesis by modulating the activation of mTOR signaling pathway (25, 74, 75), on the other it reduces protein degradation by regulating autophagy through the acetyl-coenzyme on mTOR complex 1 and by diminishing oxidative stress (76, 77).

In the current study, we have demonstrated that an anti-catabolic action of the high protein-BCAA diet ceases after 15 days (Figure 1). In agreement with our study, English et al. have demonstrated that a diet with leucine supplementation (0.06 g/kg per meal) protected against muscle loss after 7-day BR but not after 14 days in middle-aged males; showing that the beneficial effects of leucine supplementation may not be maintained through a prolonged muscle disuse (78). It is not clear why the anabolic action on protein metabolism by chronic leucine supplementation, is not maintained for prolonged periods, despite its powerful effect on acute muscle protein synthesis.

Among several potential mechanisms, including a leucine “desynchronization effect” (63) one possibility is that beyond a certain level, excess of leucine may stimulate key enzymes in BCAA catabolism (i.e., BCAA aminotransferase and branched-chain alpha-keto acid dehydrogenase) thus increasing the oxidation of serum leucine (79, 80). Moreover, a surplus in leucine intake can decrease the plasma concentration of the other EAAs, an effect called BCAA antagonism (81–83). Further investigations are needed to confirm the “desynchronization effect” hypothesis. During immobilization/BR or aging, another important mechanism explaining a reduced muscle protein synthesis, and subsequent smaller increases in lean mass in response to protein feeding, may be due to a decreased amino acid transporter expression. Among them, the large neutral amino acid transporter 1 (LAT1), which preferentially transports leucine and the other BCAAs into the cells, together with the sodium-coupled neutral amino acid transporter 2 (SNAT2), have been shown to activate mTOR signaling (84). In physiological condition, these important amino acid transporters (LAT1 and SNAT2) are sensitive to changes in nutrient status and are associated with activation of mTOR signaling and muscle protein synthesis (85). In fact, mRNA expression and protein content of LAT1 and SNAT2 are increased by EAAs and resistance exercise (86, 87). However, Drummond et al. also found that the EAA-induced increase in LAT1 and SNAT2 proteins was abolished by 7 days of BR (88). Therefore, we hypothesized that the loss of effectiveness of the high protein-BCAA diet could be associated with a decreased muscle protein synthesis by a mechanism involving reduced mTOR signaling pathway, and amino acid transporter expression, thus causing a “desynchronization effect” of leucine in the first 15 days of inactivity. Taken together, these findings suggest limited effect of increasing protein supplementation with EAAs, leucine or BCAAs in offsetting muscle loss in states of long-term BR or leg immobilization, as disuse models.

N balance has been traditionally used to estimate whole body protein balance in response to nutritional interventions (41). In this study, we have shown a consistency of the results obtained every 15 days with DXA scan measurements with those obtained with daily monitoring of the N balance. In order to compare N balance and DXA results, the average daily N balance values were calculated in the time intervals between consecutive LBM measurements. Results of N balance were consistent with those of DXA. During the first 15 days of BR, as compared with the conventional diet group, the high protein-BCAA group exhibited 42% lower LBM loss and 2.5 times greater N balance. During the remaining part of the study, no significant differences were observed between the two groups in both lean mass loss and N balance. Koyama et al. have previously compared changes in lean tissue mass measured by DXA with N balance studies in obese women, studied over two periods of treatment with a very low-energy diet (89). There was a moderate correlation between the changes in lean mass measured by the two methods (*r* = 0.40, *p* < 0.05) (89).

Limitations of our study are that only women were investigated and that sample size for each group was small (*n* = 8 per group, conventional vs. high protein-BCAA diets). Therefore, further studies should have a cross design and include larger sample sizes to investigate if there is a difference between sex that could affect the potential ergogenic benefit provided by leucine supplementation during BR. The results of the WISE—2005 study in women, were compared to those derived from many studies on long-term BR (55 days and more) in males (90–95). This is important to evaluate any sex differences in muscle atrophy and in the effectiveness of countermeasures. In a recent review, Gao and Chilibeck examined the results of previous nutritional interventions during BR for prevention of muscle loss (96). Findings were mixed, among the 11 protein/amino acid supplementation studies included in the review, 4 failed to find any beneficial effects on muscle mass (96). These discrepancies have been attributed to differences in dietary protein quantity and quality (96). Therefore, the difference between diet groups in our study may be due to differences in leucine content or in protein intake between groups (1.1 g⋅kg^–1^⋅day^–1^ vs. 1.45 g⋅kg^–1^⋅day^–1^). In fact, we believe that the apparent heterogeneity between studies (especially in short-term BR) could be the consequence of differences in the diet composition (quantity of protein), experimental models (i.e., leg immobilization vs. bed rest) and bed rest duration rather that to sex-related factors and age (Table 4) (38, 41, 78, 97–106). In addition, as referred by Stein and Blanc, baseline protein intake was different between studies failing to report a beneficial effect and those finding a positive effect (107).

**TABLE 4:** Effects of amino acid and protein supplements on muscle mass and function in different models of muscle unloading in healthy volunteers of different ages and sex.

References	Subjects	Nutritional intervention	Muscle unloading	Time	Results	Outcome^a^
Kilroe et al. (97)	Young males	High-protein intake, 1.6 g/kg/day	Leg immobilization	3 days	No effects on muscle mass (MRI) and protein synthesis (stable isotopes)	–
Dirks et al. (98)	Older males	Protein supplement, 40 g/day	Leg immobilization	5 days	No effects on muscle mass (CT) and strength	–
Backx et al. (99)	Young males	Leucine supplement, 7.5 g/day	Leg immobilization	7 days	No effects on muscle mass (CT) and strength	–
Edwards et al. (100)	Young males	Leucine supplement, 7 g/day	Leg immobilization	7 days	No effects on muscle mass (DXA), strength and protein synthesis (stable isotopes)	–
Holloway et al. (101)	Young males	Essential AA supplement, 70 g/day (BCAA 24 g/day, 50% leucine)	Leg immobilization	7 days	Preservation of muscle mass (MRI). No effects on muscle strength	+
Reidy et al. (102)	Older adults	Protein supplement, 17 g; neuromuscular electrical stimulation	Bed rest	5 days	Preservation of muscle mass (DXA); no effects on muscle strength	+
Stein et al. (41)	Young males	BCAA supplement, 12 g/day (33% leucine)	Bed rest	6 days	Preservation of nitrogen balance	+
English et al. (78)	Middle-aged male adults	Leucine supplement, 13 g/day	Bed rest	7 days	Preservation of muscle mass (DXA)	+
Arentson-Lantz et al. (103)	Older men and women	Leucine supplement, 15 g/day	Bed rest	7 days	Preservation of muscle mass (DXA); no effects on muscle strength	+
Present study	Young females	High-protein-BCAA intake, 1.6 g/kg/day (BCAA 22 g/day, 50% leucine)	Bed rest	7 days	Preservation of muscle mass (DXA) and of nitrogen balance	+
Deutz et al. (104)	Older men and women	β-hydroxy-β-methylbutyrate supplement, 3 g/day	Bed rest	10 days	Preservation of muscle mass (DXA)	+
English et al. (78)	Middle-aged male adults	Leucine, 13 g/day	Bed rest	14 days	No effects on muscle mass (DXA)	–
Rudwill et al. (105)	Young males	High-protein intake, 1.8 g/kg/day (33% whey protein)	Bed rest	21 days	No effects on muscle mass (DXA)	–
Present study	Young females	High-protein-BCAA intake, 1.6 g/kg/day (BCAA 22 g/day, 50% leucine)	Bed rest	60 days	No effects on muscle mass (DXA) or nitrogen balance	–
Owen et al. (106)	Young males	High-protein intake, 1.8 g/kg/day (33% whey protein); resistive vibration exercise	Bed rest	21 days	Preservation of muscle mass (MRI)	+
Dorfman et al. (38)	Young females	High-protein-BCAA intake, 1.6 g/kg/day (BCAA 22 g/day, 50% leucine)	Bed rest	60 days	Preservation of myocardial mass (MRI)	+

This table reports selected studies describing the effects of protein and amino acid supplements on muscle mass and function in experimental models of muscle inactivity, in young and elderly male and female subjects. The various experimental models of physical inactivity are not physiologically equivalent. Unilateral immobilization of the lower limb is associated to complete mechanical unloading of the affected muscles. In contrast, bedridden subjects maintain residual movement and muscle contraction while they are carrying out all the daily activities lying in bed. Previous observations clearly demonstrate that the anabolic effects of proteins and amino acids on muscle mass and function are directly proportional to the level of contractile activity (59, 66). It is therefore predictable that the anabolic action of amino acid and protein supplements is reduced in conditions of complete muscle unloading as in the leg immobilization model. The amino acid leucine has a particular direct effect of stimulating muscle protein synthesis compared to other amino acids (30). However, the activation of protein synthesis requires in addition the presence of all amino acids in optimal proportions. The administration of leucine causes the induction of the branched-chain α-ketoacid dehydrogenase enzyme, which irreversibly catabolizes all three BCAAs (79, 80). An excess of leucine can therefore determine a relative reduction of the other BCAAs, valine and isoleucine, resulting in a lack of stimulation of protein synthesis. The optimal action of a leucine supplementation is achieved when it is administered in addition to the other BCAAs and in combination with an adequate protein/amino acid intake (65, 83). These considerations may explain the muscle mass saving effect obtained during 7 days of leg immobilization by administering high doses of leucine in combination with the other essential amino acids including the other BCCAs. In contrast to leg immobilization studies, short-term bed rest (BR) studies (i.e., 5–10 days), which also include our present observation, consistently demonstrate a muscle-sparing effect associated with leucine, BCAA, β-hydroxy-β-methylbutyrate (a leucine metabolite) or protein supplementation. In contrast, leucine, BCAA, or protein supplementation was not associated with skeletal muscle-saving effects in middle- (i.e., 14–21 days) or long-term (i.e., 60 days) BR studies. Except for the conditions in which the nutritional supplement were associated with resistive vibration exercise or with the myocardium contractile activity. The effects of leucine, amino acid and protein supplementation on muscle mass during muscle unloading do not appear to depend on age and sex. AA, amino acids; BCAA, branched-chain amino acids; MRI, magnetic resonance imaging; CT, computed tomography; ^a^, + outcome: preservation of muscle mass and/or nitrogen balance; –outcome: no effects on muscle mass and/or nitrogen balance.

From the results of our study, it can be assumed that it is useful to administer a high-protein diet with BCAAs in the short-term BR, such as in acute illness of short duration, whereas in long-lasting pathological conditions, this nutritional approach appears useless. Nevertheless, a limitation of this study is that it has been carried out in healthy participants free of medical conditions that may exacerbate muscle loss. Prolonged immobility is harmful with rapid reductions in muscle mass, bone mineral density and impairment in other body systems. These effects are further exacerbated in individuals with critical illness. From the results of our study, we believe that if BR is absolutely recommended, as clinical intervention for a variety of health problems, a high-protein diet strategy could be helpful in mitigating short-term disuse muscle atrophy.

## Data availability statement

The raw data supporting the conclusions of this article will be made available by the authors, without undue reservation.

## Ethics statement

The studies involving human participants were reviewed and approved by the Institutional Review Boards of National Aeronautics and Space Administration, Johnson Space Center Committee for the Protection of Human Subjects, the University of California-San Diego Institutional Review Board, and Comité consultatif de Protection des Personnes dans la Recherche Biomédicale of Toulouse, France. The patients/participants provided their written informed consent to participate in this study.

## Author contributions

GB, FDG, and AM designed the study, analyzed the results, and wrote the manuscript. GB, PV, NF, and M-PB provided resources, expertise, and critically reviewed the manuscript. FM, AN, and MG prepared the figures and tables and edited the manuscript. All authors contributed to the article and approved the submitted version.
